# Structural correlations between brain magnetic resonance image‐derived phenotypes and retinal neuroanatomy

**DOI:** 10.1111/ene.16288

**Published:** 2024-05-08

**Authors:** Zihan Sun, Bing Zhang, Stephen Smith, Denize Atan, Anthony P. Khawaja, Kelsey V. Stuart, Robert N. Luben, Mahantesh I. Biradar, Thomas McGillivray, Praveen J. Patel, Peng T. Khaw, Axel Petzold, Paul J. Foster, the UK Biobank Eye and Vision Consortium

**Affiliations:** ^1^ National Institute for Health Research Biomedical Research Centre at Moorfields Eye Hospital National Health Service Foundation Trust and University College London Institute of Ophthalmology London UK; ^2^ National Clinical Research Centre for Ocular Diseases, Eye Hospital Wenzhou Medical University Wenzhou China; ^3^ Wellcome Centre for Integrative Neuroimaging (WIN Functional Magnetic Resonance Imaging Building) University of Oxford Oxford UK; ^4^ Bristol Eye Hospital University Hospitals Bristol and Weston NHS Foundation Trust Bristol UK; ^5^ Translational Health Sciences, Bristol Medical School University of Bristol Bristol UK; ^6^ Centre for Clinical Brain Sciences University of Edinburgh Edinburgh UK; ^7^ Queen Square Institute of Neurology, University College London, Department of Molecular Neurosciences Moorfields Eye Hospital and National Hospital for Neurology and Neurosurgery London UK; ^8^ Departments of Neurology and Ophthalmology and Expertise Center for Neuro‐ophthalmology Amsterdam University Medical Centre Amsterdam the Netherlands

**Keywords:** image‐derived phenotypes, magnetic resonance imaging, optical coherence tomography, retinal neurodegeneration, retinal thickness

## Abstract

**Background and purpose:**

The eye is a well‐established model of brain structure and function, yet region‐specific structural correlations between the retina and the brain remain underexplored. Therefore, we aim to explore and describe the relationships between the retinal layer thicknesses and brain magnetic resonance image (MRI)‐derived phenotypes in UK Biobank.

**Methods:**

Participants with both quality‐controlled optical coherence tomography (OCT) and brain MRI were included in this study. Retinal sublayer thicknesses and total macular thickness were derived from OCT scans. Brain image‐derived phenotypes (IDPs) of 153 cortical and subcortical regions were processed from MRI scans. We utilized multivariable linear regression models to examine the association between retinal thickness and brain regional volumes. All analyses were corrected for multiple testing and adjusted for confounders.

**Results:**

Data from 6446 participants were included in this study. We identified significant associations between volumetric brain MRI measures of subregions in the occipital lobe (intracalcarine cortex), parietal lobe (postcentral gyrus), cerebellum (lobules VI, VIIb, VIIIa, VIIIb, and IX), and deep brain structures (thalamus, hippocampus, caudate, putamen, pallidum, and accumbens) and the thickness of the innermost retinal sublayers and total macular thickness (all *p* < 3.3 × 10^−5^). We did not observe statistically significant associations between brain IDPs and the thickness of the outer retinal sublayers.

**Conclusions:**

Thinner inner and total retinal thicknesses are associated with smaller volumes of specific brain regions. Notably, these relationships extend beyond anatomically established retina–brain connections.

## INTRODUCTION

The projected increase in dementia and mild cognitive impairment suggests an impending health care crisis [1, 2]. Consequently, identifying lifestyle or therapeutic interventions that retard or arrest cognitive decline and dementia is a priority [3]. However, efforts have been thwarted by failures in clinical trials and adverse effects of treatment [4, 5, 6]. One theory is that interventions are introduced too late in the disease to offer meaningful benefits [7]. Even the most well‐resourced health care systems are not able to support community screening by magnetic resonance imaging (MRI). The choice of appropriate outcome measures for clinical trials remains the subject of debate [8].

The eye offers well‐established insights into brain structure and function. A thin retina is a well‐recognized corollary of Alzheimer disease (AD) [9]. We have found that a thinner retina is associated with weaker current cognitive performance and accelerated cognitive decline [10]. Similarly, people with thinner retinas are more likely to develop dementia [11]. In the same cohort, we have also shown that inner and total retinal thicknesses are correlated with total brain, grey and white matter, and occipital lobe volumes. The macular ganglion cell complex (GCC) and total retinal thicknesses show a significant correlation with hippocampal volume, hinting at a potential role for retinal imaging in identifying those people with an increased risk of cognitive decline [12]. Optical coherence tomography (OCT) offers a quick, widely available, noninvasive, reproducible tool to measure retinal layer thicknesses, a structural biomarker for cognitive health. This may assist the identification of a risk‐enriched cohort of participants for clinical trials. We used data from UK Biobank to explore the relationship between retinal layer thicknesses [13, 14] and brain MRI‐derived structural phenotypes [15].

## METHODS

### Study population

UK Biobank is a prospective population‐based multicentre cohort study of >500,000 participants residing in the UK and registered with the National Health Service. Participants aged 37–73 years were initially recruited between 2006 and 2010. The North West Multicentre Research Ethics Committee approved the study in accordance with the principles of the Declaration of Helsinki. Study protocols have been published online (https://www.ukbiobank.ac.uk/media/gnkeyh2q/study‐rationale.pdf). In brief, participants answered a wide range of touchscreen questionnaires covering demographic, socioeconomic, and lifestyle information along with comprehensive physical measurements. A subset of UK Biobank participants underwent detailed ophthalmic assessments, including retinal imaging, at their initial assessment visit (2009–2010) and follow‐up (2012–2013) [16]. In 2014, UK Biobank launched the imaging enhancement study, the world's largest multimodal imaging study aiming to include MRI of the brain, heart, and abdomen, whole‐body dual‐energy X‐ray absorptiometry and carotid Doppler ultrasound for up to 100,000 participants. Notably, invitations for this imaging enhancement study were extended to individuals regardless of whether they had prior ophthalmic assessments [17].

### Retinal imaging

Macula‐centred OCT was performed using the three‐dimensional (3D) OCT‐1000 Mk2 device (Topcon, Tokyo, Japan). Image acquisition was performed under mesopic conditions, without pupillary dilation, using the 3D macular volume scan (512 horizontal A scans per B scan; 128 B scans in a 6 × 6 mm^2^ raster pattern). Images from both eyes, where available, were used. We first included participants who had retinal imaging at the same baseline assessment (2009–2010) as when they completed their touchscreen questionnaires. For participants without available OCT images at the baseline visit, we used the OCT data at their follow‐up visit (2012–2013) for analysis. OCT‐derived retinal thicknesses were estimated using the Topcon Advanced Boundary Segmentation Tool (TABS), software providing automated segmentation of retinal sublayers using dual‐scale gradients [13]. TABS provides additional metadata for each image to establish scan quality based on segmentation error, movement artefact, and poor quality. Quality control (QC) of OCTs was performed as previously described [18].

### Brain MRI

Brain MRI data were acquired using a Siemens Skyra 3T scanner (Siemens Healthcare, Erlangen, Germany) using a 32‐channel radiofrequency receive head coil (see http://www.fmrib.ox.ac.uk/ukbiobank/protocol/V4_23092014.pdf; https://biobank.ndph.ox.ac.uk/showcase/ukb/docs/brain_mri.pdf). Structural imaging data were quality checked and processed to provide imaging‐derived phenotypes (IDPs) as described [15, 19]. IDPs (*n* = 153) used in this study were 139 regional grey matter volumes (GMVs) and 14 subcortical structures' volumes, derived using parcellations from the Harvard‐Oxford Cortical and Subcortical Atlases (https://fsl.fmrib.ox.ac.uk/fsl/fslwiki/Atlases) and the Diedrichsen Cerebellar Atlas (http://www.diedrichsenlab.org/imaging/propatlas.htm). Volumetric grey matter IDPs in 139 regions of interest were generated from using FAST (FMRIB's Automated Segmentation Tool) [20]; subcortical structures volumes were modelled using FIRST (FMRIB's Integrated Registration and Segmentation Tool) [21]. We normalized all the raw IDPs to head size using the T1‐based head size scaling factor (UK Biobank data field 25,000). The complete list of selected IDPs and their UK Biobank data field IDs are summarized in eTable 1.

Assessments of age, sex, ethnicity, education level, Townsend deprivation index, mean arterial pressure, body mass index, smoking status, alcohol intake, self‐reported diabetes mellitus and use of antihypertension medications, intraocular pressure, spherical equivalent, and glaucoma diagnosis are provided in eMethods.

### Inclusion and exclusion criteria

Among participants with both OCT and MRI data available, those who met the following criteria were excluded, consistent with the OSCAR‐IB criteria [22, 23], from the study to avoid local eye pathology masking the more subtle effects of neurodegeneration: (i) poor QC, (ii) both eyes' visual acuity worse than 0.5 logarithms of the minimum angle of resolution (logMAR), (iii) either eye's corneal compensated intraocular pressure (IOPcc) < 6 mmHg or > 24 mmHg, (iv) self‐reported history of glaucoma (or glaucoma laser or glaucoma surgery), (v) an International Classification of Diseases (ICD)‐9 or ICD‐10 code for any types of glaucoma before or up to 1 year after baseline assessment (see eMethods), or (vi) self‐reported neurological conditions (eTable 2). We did not exclude participants with dementia in the main analysis, to avoid truncating the distribution of brain IDPs that would have affected the discovery power.

### Statistical analysis

Participant‐level retinal thicknesses were calculated as the mean of right and left eye values to minimize measurement errors between eyes. If data were available only for one eye, that value was used for analysis. We did not derive participant‐level brain IDPs by averaging left and right hemispheric data, because the laterality information is important for some neurological conditions [24, 25]. We first examined 1530 (10 × 153 = 1530) pairs of crude associations between retinal thicknesses and brain IDPs, where the retinal metrics were regarded as independent variables and brain IDPs as dependent variables using univariable linear regression. In the multivariable linear regression model, we adjusted for age, sex, imaging site, time lapse between OCT and MRI, education level, mean arterial pressure, body mass index, smoking status, alcohol intake, diabetes mellitus, and spherical equivalent refraction [18, 26, 27, 28, 29, 30, 31, 32, 33, 34, 35]. Notably, spherical equivalent serves as a proxy for axial length—an unmeasured parameter in the UK Biobank dataset—thereby offering an indirect measure of eyeball size. This adjustment was made to account for individual anatomical variations, aligning with the common practice of adjusting for head size when evaluating brain volumetrics. All analyses were conducted in R (version 4.1.0, R Foundation for Statistical Computing). *p* < 3.268 × 10^−5^ (0.05/1530 = 3.268 × 10^−5^) following Bonferroni correction was considered statistically significant.

## RESULTS

There were 6650 participants with usable brain MRI and OCT imaging data. It is important to note that OCT and MRI scans took place during separate visits. OCT data were collected in two periods—either 2009–2010 or 2013–2014—whereas brain MRI data spanned from 2014 to 2020. The mean time lapse between OCT and MRI scans was 6.05 years (median = 6, interquartile range [IQR] = 4, range = 1–11). Of these, 204 people were excluded because of visual acuity worse than 0.5 logMAR, IOPcc of <6 mmHg or >24 mmHg, or a self‐reported history of glaucoma or neurological conditions. This left 6446 participants aged 40–75 years (mean = 57, median = 58, IQR = 12) for analysis (eFigure 1). Baseline characteristics are detailed in Table 1.

**TABLE 1:** Demographic, systemic, and ocular characteristics of the study population.

Characteristics	*n*	Mean ± SD or *n* (%)
Age, years	6446	57.12 ± 7.73
Sex	6446	
Male		3192 (49.52)
Female		3254 (50.48)
Ethnicity	6442	
White		6233 (96.76)
Asian		89 (1.38)
Black		44 (0.68)
Other/mixed/unknown		76 (1.18)
Townsend deprivation index	6438	−1.96 ± 2.62
Education level	6446	
O level or less		1221 (18.94)
A level or professional quantifications		1608 (24.95)
University degree		3335 (51.74)
Prefer not to say		282 (4.37)
Body mass index, kg/m^2^	6446	26.51 ± 4.18
Mean arterial blood pressure, mmHg	6444	98.48 ± 10.33
Smoking status	6442	
Current		381 (5.92)
Previous		2140 (33.22)
Never		3913 (60.74)
Prefer not to answer		8 (0.12)
Alcohol intake, g/week^a^	6442	91.28 (126.91)
Self‐reported diabetes	6446	
Yes		347 (5.38)
No		6099 (94.62)
SE, diopters	6446	−0.09 ± 1.96
IOPcc, mmHg	6351	15.72 ± 3.02
Retinal OCT metrics	6446	
mRNFL thickness, μm		31.19 ± 4.99
GCIPL thickness, μm		71.40 ± 6.43
GCC thickness, μm		102.59 ± 7.48
INL thickness, μm		32.46 ± 2.08
INL‐ELM thickness, μm		80.40 ± 5.62
INL‐RPE thickness, μm		142.80 ± 6.79
ELM‐ISOS thickness, μm		23.70 ± 1.36
ISOS‐RPE thickness, μm		38.75 ± 3.32
RPE thickness, μm		24.89 ± 2.48
Total macular thickness, μm		277.90 ± 11.74

Abbreviations: ELM‐ISOS, external limiting membrane–inner segment outer segment; GCC, ganglion cell complex; GCIPL, ganglion cell–inner plexiform layer; INL, inner nuclear layer; INL‐ELM, inner nuclear layer–external limiting membrane; INL‐RPE, inner nuclear layer–retinal pigment epithelium; IOPcc, corneal compensated intraocular pressure; ISOS‐RPE, inner segment outer segment–retinal pigment epithelium; mRNFL, macular nerve fibre layer; OCT, optical coherence tomography; RPE, retinal pigment epithelium; SE, spherical equivalent.

^a^ Alcohol intake quantity is presented as median (interquartile range) due to its right‐skewed distribution.

### Univariable associations between retinal metrics and brain IDPs

**FIGURE 1:**
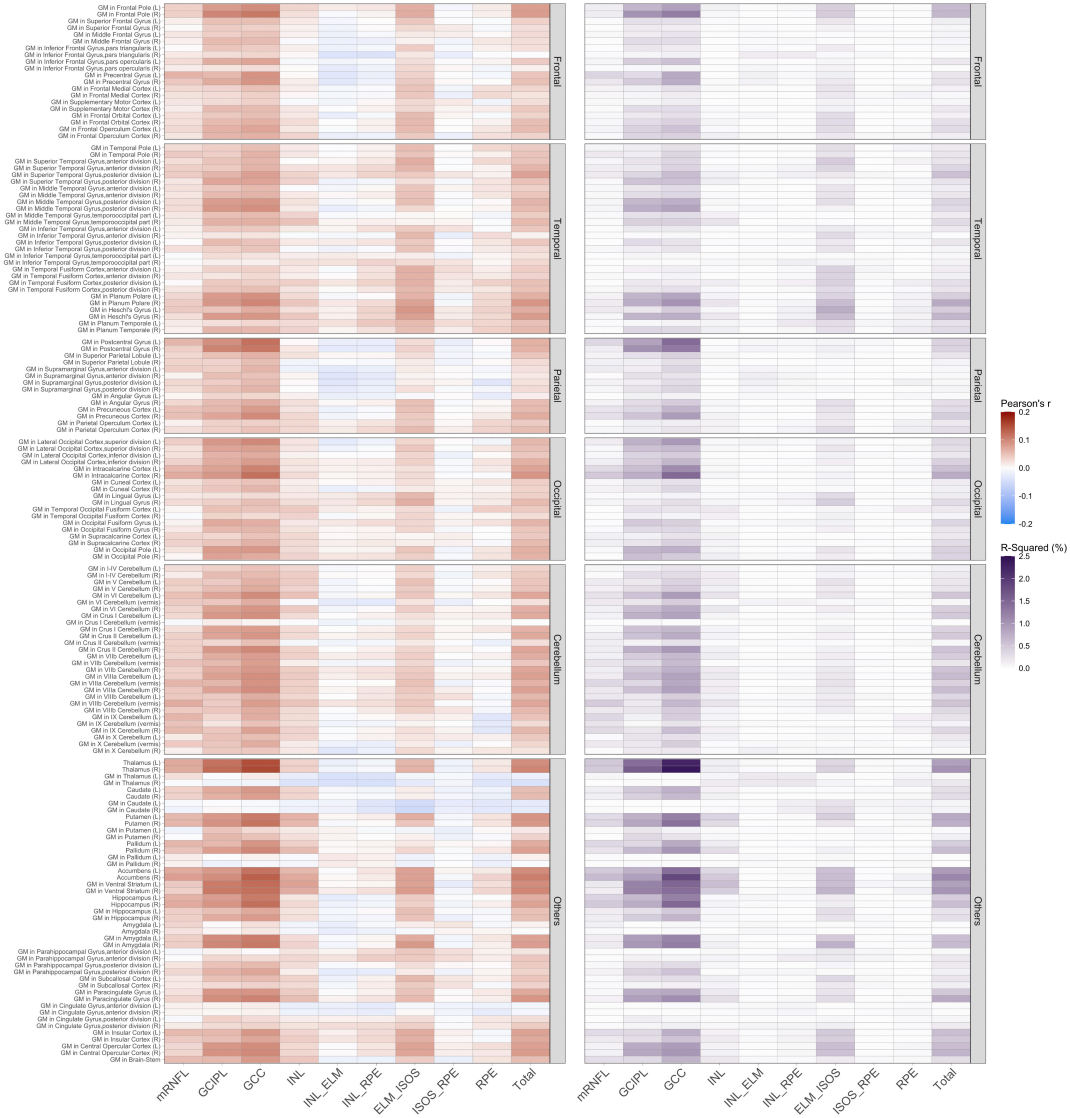
Illustrative heatmaps of pairwise univariate correlations between 10 retinal metrics and 153 brain magnetic resonance image derived phenotypes (IDPs). The left panel is a heatmap presenting Pearson correlation coefficients (*r*), which measure the correlations between 10 retinal metrics and 153 brain IDPs. Cells in red represent a positive correlation (Pearson *r* > 0) and in blue represent a negative correlation (Pearson *r* < 0), where darker colour indicates a stronger correlation (larger absolute value of Pearson *r*). The right panel is a heatmap plotting the *R* ^2^ of pairwise univariate linear regression analysis of the association between retinal metrics and brain IDPs. A darker colour represents a higher *R* ^2^ value, indicating better goodness‐of‐fit of the model. ELM_ISOS, external limiting membrane–inner segment outer segment; GCC, ganglion cell complex; GCIPL, ganglion cell–inner plexiform layer; GM, grey matter; INL, inner nuclear layer; INL_ELM, inner nuclear layer–external limiting membrane; INL_RPE, inner nuclear layer–retinal pigment epithelium; ISOS_RPE, inner segment outer segment–retinal pigment epithelium; L, left hemisphere; mRNFL, macular retinal nerve fibre layer; R, right hemisphere; RPE, retinal pigment epithelium; Total, total macular thickness.

**FIGURE 2:**
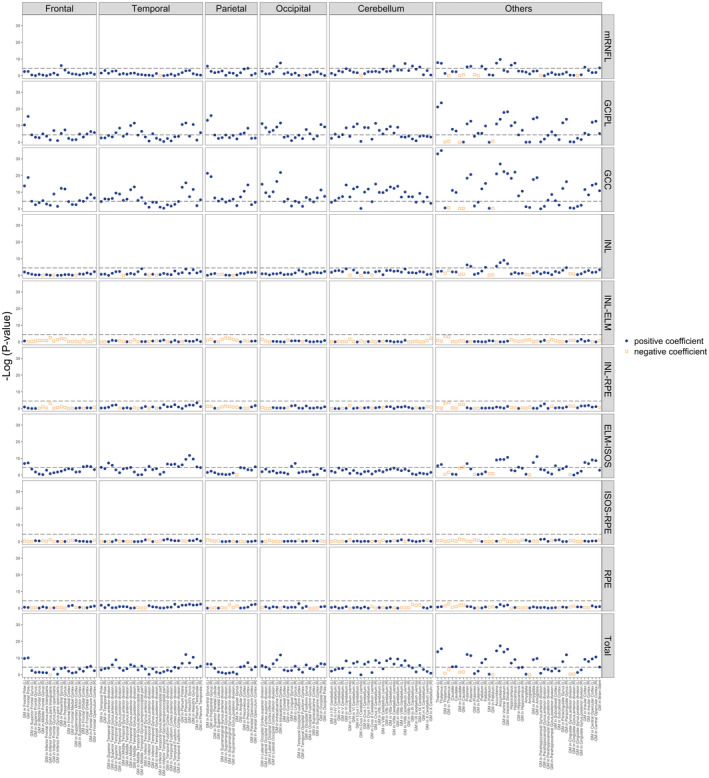
Visual representation of the results of pairwise univariate association tests between 10 retinal metrics and 153 brain magnetic resonance image derived phenotypes. Univariable, pairwise linear regressions were performed using data from *n* = 6446 participants. Each datapoint represents a single retina–brain association. Blue circles indicate positive regression coefficients; orange squares indicate negative regression coefficients. We followed the convention for Manhattan plots and plotted −log10 (*p* values) on the y‐axis. The dashed horizontal line indicates the −log10 (*p*) threshold after Bonferroni correction is applied, and all associations above this line are considered statistically significant at *p* < 3.268 × 10^−5^ (corresponding to a −log10 [*p*] of 4.4857). ELM‐ISOS, external limiting membrane–inner segment outer segment; GCC, ganglion cell complex; GCIPL, ganglion cell–inner plexiform layer; GM, grey matter; INL, inner nuclear layer; INL‐ELM, inner nuclear layer–external limiting membrane; INL‐RPE, inner nuclear layer–retinal pigment epithelium; ISOS‐RPE, inner segment outer segment–retinal pigment epithelium; L, left hemisphere; mRNFL, macular retinal nerve fibre layer; R, right hemisphere; RPE, retinal pigment epithelium; Total, total macular thickness.

We first performed the univariate, pairwise association analyses between 10 retinal metrics and 153 brain IDPs, where Pearson's correlation *r* was also calculated (Figures 1 and 2). We observed a range of statistically significant associations across different brain regions. The strongest of these associations was observed between GCC and the right thalamus (*r =* 0.154, *p =* 1.14 × 10^−35^). The full set of 1530 pairs of univariable associations are given in eTable 3. Of the 313 pairs of statistically significant correlations, most were positive (*n* = 312), suggesting that a thinner retina may indicate regional brain atrophy.

### Multivariable associations between retinal metrics and brain IDPs

**FIGURE 3:**
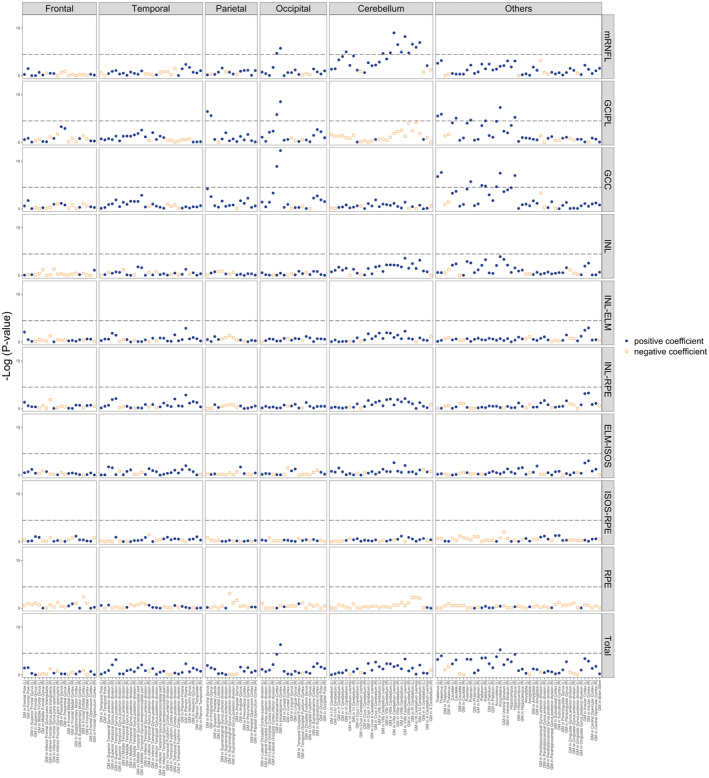
Visual representation of the results of pairwise multivariable association tests between 10 retinal metrics and 153 brain magnetic resonance image derived phenotypes. Multivariable linear regressions were performed using data from *n* = 6421 participants, adjusting for age, sex, imaging site, the time lapse between optical coherence tomography and magnetic resonance image scan, education level, mean arterial pressure, body mass index, smoking status, alcohol intake, diabetes mellitus, and spherical equivalence. Each datapoint represents a single retina–brain association. Blue circles indicate positive regression coefficients; orange squares indicate negative regression coefficients. We followed the convention for Manhattan plots and plotted −log10 (*p* values) on the y‐axis. The dashed horizontal line indicates the −log10 (*p*) threshold after Bonferroni correction is applied, and all associations above this line are considered statistically significant at *p* < 3.268 × 10^−5^ (corresponding to a −log10 [*p*] of 4.4857). ELM‐ISOS, external limiting membrane–inner segment outer segment; GCC, ganglion cell complex; GCIPL, ganglion cell–inner plexiform layer; GM, grey matter; INL, inner nuclear layer; INL‐ELM, inner nuclear layer–external limiting membrane; INL‐RPE, inner nuclear layer–retinal pigment epithelium; ISOS‐RPE, inner segment outer segment–retinal pigment epithelium; L, left hemisphere; mRNFL, macular retinal nerve fibre layer; R, right hemisphere; RPE, retinal pigment epithelium; Total, total macular thickness.

**FIGURE 4:**
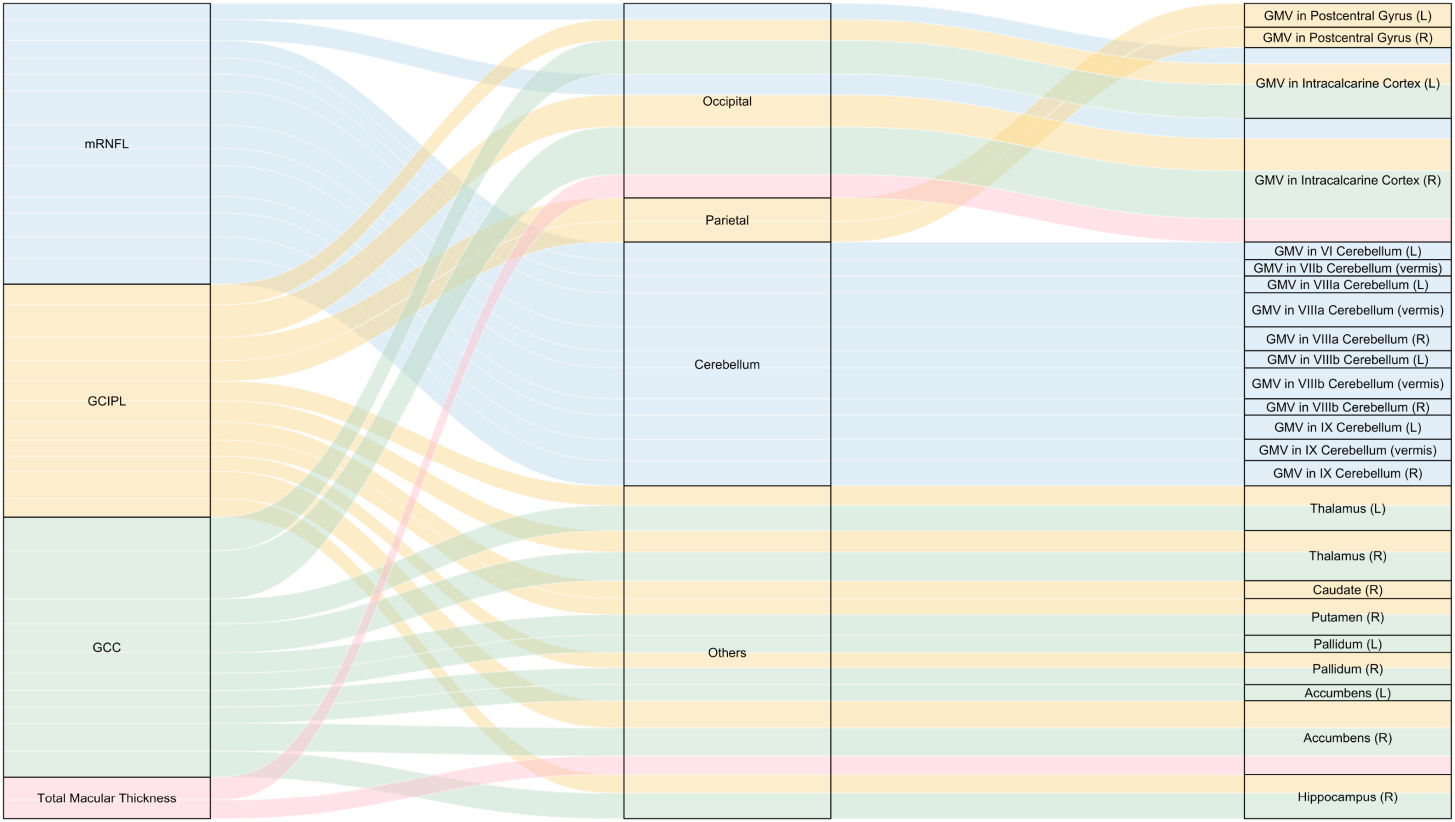
Alluvial diagram illustrating the statistically significant retina–brain associations. For clarity, only statistically significant associations identified by multivariable regression (Table 2) are shown in this diagram. Covariates adjusted in these multivariable models include age, sex, imaging site, the time lapse between optical coherence tomography scan and magnetic resonance image scan, education level, mean arterial pressure, body mass index, smoking status, alcohol intake, diabetes mellitus, and spherical equivalence. Each stream in the diagram corresponds to a datapoint (retina–brain association) shown in Figure 3 falling above the Bonferroni‐corrected threshold line. The width of each stream is proportional to the partial *R* ^2^, as shown in Table 2. GCC, ganglion cell complex; GCIPL, ganglion cell–inner plexiform layer; GMV, grey matter volume; L, left hemisphere; mRNFL, macular retinal nerve fibre layer; R, right hemisphere.

We then performed multivariable pairwise association analysis, controlling for covariates as outlined in Methods. A complete list of multivariable pairwise associations is shown in eTable 4. Most of the correlations in the univariate analysis were attenuated, leaving 36 pairs of significant associations (shown in Figure 3). Table 2 and Figure 4 elucidate the statistically significant associations between retinal layer thicknesses and brain IDPs. Thinner macular retinal nerve fibre layer (mRNFL) thickness was predominantly associated with lower GMV in different segments of the cerebellum, including the left lobule VI, vermis of lobule VIIb, and lobules VIIIa, VIIIb, and IX. Additionally, a notable association was observed in the occipital lobe–bilateral intracalcarine cortex, with all *p* values < 3.268 × 10^−5^. Ganglion cell–inner plexiform layer (GCIPL) thickness displayed strong correlations with GMV in both the occipital lobe (specifically the bilateral intracalcarine cortex) and the parietal lobe (bilateral postcentral gyri), and with volumes of several subcortical structures including the bilateral thalami, right caudate, right putamen, right pallidum, right accumbens, and right hippocampus (all with *p* values < 3.268 × 10^−5^). GCC thickness showed significant associations especially with GMV in bilateral intracalcarine cortex in the occipital lobe, as well as with volumes of subcortical structures such as bilateral thalami, right putamen, bilateral pallidum, bilateral accumbens, and right hippocampus (all with *p* values < 3.268 × 10^−5^). A thinner total macular thickness was linked to a lower GMV in right intracalcarine cortex (*p* = 4.87 × 10^−7^) and a smaller volume of the right accumbens (*p* = 5.78 × 10^−6^).

**TABLE 2:** Multivariable analysis of associations between retinal layer thicknesses and brain magnetic resonance IDPs.

Retinal Metric, μm	Lobe	Brain IDP, mm^3^	*β* coefficient (95% CI)	*p*	Partial *R* ^2^, %	*R* ^2^, %
mRNFL thickness	Occipital	GMV in intracalcarine cortex (L)	0.060 (0.033–0.088)	2.10 × 10^−5^	0.27	3.0
mRNFL thickness	Occipital	GMV in intracalcarine cortex (R)	0.068 (0.040–0.095)	1.77 × 10^−6^	0.34	3.9
mRNFL thickness	Cerebellum	GMV in VI cerebellum (L)	0.058 (0.033–0.084)	1.01 × 10^−5^	0.29	15.6
mRNFL thickness	Cerebellum	GMV in VIIb cerebellum (vermis)	0.058 (0.031–0.084)	2.17 × 10^−5^	0.27	11.8
mRNFL thickness	Cerebellum	GMV in VIIIa cerebellum (L)	0.057 (0.031–0.083)	1.57 × 10^−5^	0.27	15.3
mRNFL thickness	Cerebellum	GMV in VIIIa cerebellum (vermis)	0.082 (0.056–0.109)	1.03 × 10^−9^	0.56	12.9
mRNFL thickness	Cerebellum	GMV in VIIIa cerebellum (R)	0.067 (0.041–0.093)	3.08 × 10^−7^	0.39	17.9
mRNFL thickness	Cerebellum	GMV in VIIIb cerebellum (L)	0.060 (0.033–0.087)	1.13 × 10^−5^	0.28	10.5
mRNFL thickness	Cerebellum	GMV in VIIIb cerebellum (vermis)	0.080 (0.053–0.107)	6.21 × 10^−9^	0.50	9.5
mRNFL thickness	Cerebellum	GMV in VIIIb cerebellum (R)	0.057 (0.031–0.084)	1.71 × 10^−5^	0.27	14.3
mRNFL thickness	Cerebellum	GMV in IX cerebellum (L)	0.070 (0.043–0.096)	2.49 × 10^−7^	0.39	12.2
mRNFL thickness	Cerebellum	GMV in IX cerebellum (vermis)	0.067 (0.040–0.095)	1.12 × 10^−6^	0.35	7.9
mRNFL thickness	Cerebellum	GMV in IX cerebellum (R)	0.071 (0.045–0.098)	1.08 × 10^−7^	0.42	13.2
GCIPL thickness	Parietal	GMV in postcentral gyrus (L)	0.068 (0.042–0.095)	3.57 × 10^−7^	0.39	20.9
GCIPL thickness	Parietal	GMV in postcentral gyrus (R)	0.062 (0.036–0.089)	2.65 × 10^−6^	0.33	22.0
GCIPL thickness	Occipital	GMV in intracalcarine cortex (L)	0.071 (0.042–0.100)	1.51 × 10^−6^	0.34	3.1
GCIPL thickness	Occipital	GMV in intracalcarine cortex (R)	0.087 (0.059–0.116)	3.19 × 10^−9^	0.53	4.1
GCIPL thickness	Others	Volume of thalamus (L)	0.061 (0.035–0.086)	3.12 × 10^−6^	0.32	25.4
GCIPL thickness	Others	Volume of thalamus (R)	0.063 (0.038–0.089)	1.30 × 10^−6^	0.35	25.3
GCIPL thickness	Others	Volume of caudate (R)	0.065 (0.036–0.093)	8.83 × 10^−6^	0.29	6.1
GCIPL thickness	Others	Volume of putamen (R)	0.060 (0.032–0.087)	2.25 × 10^−5^	0.26	12.9
GCIPL thickness	Others	Volume of pallidum (R)	0.060 (0.032–0.089)	3.23 × 10^−5^	0.25	6.9
GCIPL thickness	Others	Volume of accumbens (R)	0.074 (0.047–0.100)	5.21 × 10^−8^	0.45	19.1
GCIPL thickness	Others	Volume of hippocampus (R)	0.064 (0.036–0.091)	5.98 × 10^−6^	0.30	12.9
GCC thickness	Occipital	GMV in intracalcarine cortex (L)	0.077 (0.052–0.102)	1.29 × 10^−9^	0.55	3.3
GCC thickness	Occipital	GMV in intracalcarine cortex (R)	0.091 (0.066–0.115)	6.05 × 10^−13^	0.78	4.4
GCC thickness	Others	Volume of thalamus (L)	0.058 (0.036–0.080)	1.68 × 10^−7^	0.41	25.5
GCC thickness	Others	Volume of thalamus (R)	0.062 (0.040–0.084)	2.32 × 10^−8^	0.47	25.4
GCC thickness	Others	Volume of putamen (R)	0.057 (0.034–0.081)	2.05 × 10^−6^	0.34	13.0
GCC thickness	Others	Volume of pallidum (L)	0.055 (0.030–0.080)	1.24 × 10^−5^	0.28	4.5
GCC thickness	Others	Volume of pallidum (R)	0.053 (0.029–0.078)	1.68 × 10^−5^	0.27	7.0
GCC thickness	Others	Volume of accumbens (L)	0.050 (0.027–0.072)	2.33 × 10^−5^	0.26	17.2
GCC thickness	Others	Volume of accumbens (R)	0.064 (0.041–0.087)	3.39 × 10^−8^	0.46	19.1
GCC thickness	Others	Volume of hippocampus (R)	0.064 (0.040–0.087)	1.05 × 10^−7^	0.42	13.0
Total macular thickness	Occipital	GMV in intracalcarine cortex (R)	0.063 (0.030–0.088)	4.87 × 10^−7^	0.38	4.0
Total macular thickness	Others	Volume of accumbens (R)	0.052 (0.020–0.075)	5.78 × 10^−6^	0.30	19.0

*Note*: In this table, only statistically significant results are presented. Retinal layer thicknesses serve as the independent variables, with brain IDPs as the dependent variables. The multivariable regression models were adjusted for factors including age, sex, imaging site, the time lapse between OCT and magnetic resonance image, education level, mean arterial pressure, body mass index, smoking status, alcohol intake, diabetes, and spherical equivalence. To clarify, the presented *p*‐values are raw and have not been Bonferroni‐corrected. Standardized *β* coefficients are presented as per SD difference of retinal thickness in per SD difference of corresponding brain structure volume. Raw regression coefficient (1 μm–1 mm^3^) values are provided in the supplementary materials (eTable 4). The partial *R* ^2^ quantifies the percentage of variance in brain IDP attributable to a specific OCT‐derived retinal layer thickness, whereas the overall *R* ^2^ indicates the variance explained by all predictors in the model.

Abbreviations: CI, confidence interval; GMV, grey matter volume; IDP, imaging‐derived phenotype; L, left hemisphere; mRNFL, macular retinal nerve fibre layer; OCT, optical coherence tomography; R, right hemisphere.

Noting that age is the major source of covariation between retina and brain measures (see partial *R* ^2^ of each predictors in the model, eTable 5), we further adjusted for age squared (age^2^) in the supplementary analysis (eTable 6, eFigure 2). Additional adjustment for age^2^ did not meaningfully change the effect estimates between retinal metrics and brain IDPs. In addition, we conducted another sensitivity analysis (eTable 7) to account for the influence of antihypertensive medication use. Although the results of this analysis revealed slight variations, they remained overall consistent with our primary findings. We also explored retina–brain associations according to cerebral vascular supply territories (eFigure 3), where no significant effect was observed.

In the sensitivity analysis (eTable 8), participants with a self‐reported history of multiple sclerosis (*n* = 9), Parkinson disease (*n* = 5), dementia/AD/cognitive impairment (*n* = 1), or stroke (*n* = 69) were excluded, resulting in a subset of 6362 eligible participants for analysis. The majority (32/36) of the initially identified associations remained statistically significant. This outcome suggests that the observed retina–brain associations may not be solely driven by manifest neurodegenerative processes; instead, alternative factors, including potential contributions from neurodevelopmental aspects, might influence the observed patterns. Specifically, associations involving the GCC, GCIPL with the putamen, and pallidum no longer reached statistical significance in this sensitivity analysis.

## DISCUSSION

To our best knowledge, this is the largest study to examine the structural correlations between retinal OCT and brain MRI measures. We have identified regional volumetric MRI measures of the occipital lobe (intracalcarine cortex), the parietal lobe (postcentral gyrus), the cerebellum (lobules VI, VIIb, VIIIa, VIIIb, and IX), and subcortical structures (thalamus, hippocampus, caudate, putamen, pallidum, and accumbens) that are associated with inner (RNFL to GCIPL) and total cross‐sectional retinal thicknesses. In this study, we did not observe significant associations between the outer retinal layer thicknesses and brain IDPs, suggesting that the outer retina may be less relevant than the inner retina in assessing neurodegeneration occurring in the brain.

Consistent with previous findings, we observed an association between the inner retina and thalamus in both hemispheres [36, 37]. Data from the Rotterdam study showed that a thinner ganglion cell layer is associated with lower grey matter density in the thalamus, which could partially explain the associations we observed with thalamus volume, as changes in contrast in some thalamus edge voxels (e.g., due to changes in grey matter density) may result in apparent changes in volume. In humans, signals originating from the retina synapse in the posterior lateral geniculate nucleus in the thalamus then project onto the occipital lobes [38], demonstrating the vital role of the thalamus in relaying sensory information from the periphery to the cerebral cortex [39].

In line with previous reports [12, 36, 37, 40, 41], we found that thinner mRNFL, GCIPL, and GCC were associated with smaller GMVs of the intracalcarine cortex bilaterally, and total macular thickness was correlated with right intracalcarine cortex. The relationship between retina and primary visual cortex (V1) is widely documented in neuroimaging studies among patients with ocular and neurological diseases, and among healthy individuals [37, 42, 43, 44, 45, 46, 47]. Thus, detecting these expected associations increases our confidence about the validity of two novel findings in this exploratory study.

First, we found that thinner GCIPL was associated with smaller GMVs of the postcentral gyrus in the parietal lobes bilaterally. The postcentral gyrus contains the primary somatosensory cortex (S1), responsible for touch, pressure, temperature, and pain perception [48]. One possible explanation is that somatosensation and vision are closely related systems. Although the structural basis of any connection is unclear, existing evidence does indicate a solid physiological link between S1 and V1 in congenital blindness, with the phenomenon of enhanced tactile skills in blind individuals [49, 50, 51]. Even in normally sighted individuals, some level of tactile discrimination is provided by the visual cortex [52]. Further studies are needed to validate and uncover the underlying mechanisms of this novel finding.

Second, there was a consistent, significant association between mRNFL thickness and GMV in the posterior cerebellum, in lobules VI–IX. The cerebellum coordinates unconscious regulation of balance and muscle tone, as well as coordination of voluntary movements. The flocculonodular lobe (X) coordinates vestibulo‐ocular reflexes and eye movements, although we did not identify any dimensional relationship between lobe X and retinal metrics [53]. The anterior cerebellum (lobules I–V) provides sensorimotor proprioceptive function, primarily receiving input from the spinal cord, with a second representation in lobule VIII in the posterior lobe [54]. Although we did not identify any association with lobules I–V, we did detect significant associations between lobules VIIIa and VIIIb in the right and left hemispheres, the vermis, and mRNFL thickness, suggesting significant integration of the visual system and the sensorimotor cerebellum. In addition, we identified a relationship between lobule VI (left) and VIIb (vermis). These regions are believed to contribute to higher level processes, such as cognitive and emotional functions [55, 56]. Patients with cerebellar damage often present with the cerebellar motor syndrome of dysmetria, dysarthria, and ataxia, yet cerebellar lesions can also result in cerebellar cognitive affective syndrome, including executive, visual–spatial, and linguistic impairments, and affective dysregulation. It has been hypothesized that lobules VI and VII of the posterior lobe comprise the “cognitive cerebellum” [57]. The posterior vermis is the anatomical substrate of the limbic cerebellum. It is interesting to note that cognitive impairments occur when posterior lobe lesions affect lobules VI and VII (including crus I, crus II, and lobule VIIB), disrupting cerebellar modulation of cognitive loops with cerebral association cortices, whereas neuropsychiatric disorders manifest when vermis lesions deprive cerebrocerebellar limbic loops of cerebellar input [58]. Neuroimaging studies have suggested lateralization of cerebellar function with language served on the right‐hand side and spatial awareness on the left, consistent with our finding of an association between mRNFL thickness and GMV in left lobule VI [59]. Finally, we identified significant associations with lobule IX (left, right, and vermis). This region of the cerebellum is considered essential to visual guidance of movement [60].

The study design allows us to identify associations but does not permit the establishment of direct anatomical connections between the retina and specific brain regions. Shared susceptibility to pathological processes such as genetic architecture, environmental toxins, or other extrinsic factors might also cause volume loss in specific brain regions and simultaneously affect the retina. Inclusion of diffusion tensor imaging and functional MRI in future studies will help bridge the gap between structural correlations and anatomical or functional connections, providing a more comprehensive understanding of the relationships we have observed.

Extending our prior analysis based on data from 2131 UK Biobank participants [12], the current results concur with the previously observed association between hippocampal volume and inner retinal thickness, now supported by a larger sample (*n* = 6446). This aligns with findings from the Rotterdam study, the Rhineland study, and others [36, 40, 61, 62, 63, 64]. Hippocampal atrophy is one of the hallmark features of overt AD and mild cognitive impairment, and is a risk factor for future AD in cognitively intact elderly people [65, 66, 67]. Regarding the crucial role of the hippocampus in cognition [68, 69], the present data support the concept that inner retinal measures may serve as a structural biomarker for early cognitive decline, possibly providing people with opportunities to change their lifestyles, and facilitate risk‐stratified enrolment into drug trials to delay or avert the onset of dementia. Although our findings are certainly promising, they do come with notable limitations. Specifically, thinning of RNFL or GCIPL is not unique to AD; it has also been observed across various other central nervous system disorders. Therefore, these retinal changes should be considered within a broader diagnostic framework, because their standalone value as definitive indicators of AD is limited. Furthermore, our research into the visual pathway, particularly the thalamus and intracalcarine cortex, provides insights into the visual symptoms often seen in AD [70].

Interestingly, we also found that thinner GCIPL, GCC, and total macular thicknesses were associated with smaller volumes of basal ganglia structures. The basal ganglia are a group of subcortical nuclei responsible primarily for motor control (including eyes) and other roles such as motor learning, executive functions and behaviours, and emotions [71]. It is noteworthy that the strongest association within the basal ganglia structures was the nuclear accumbens, part of the limbic system. The accumbens is considered a node between the executive control network and reward network through its projection to the frontal cortex and limbic pathway [72]. It is, therefore, integral to several cognitive and emotional functions. Abnormalities within the accumbens have been linked to numerous underlying psychiatric conditions, such as schizophrenia, drug addiction, depression, and obsessive–compulsive disorder [73, 74, 75]. Thus, our findings suggest the potential of retinal structural measures as biomarkers for psychiatric disorders [76].

The strengths of this study include the large sample size, quantitative, comprehensive, and region‐specific assessments of the retina and brain structures, and extensive data on covariates. Our study does have limitations. First, participants in UK Biobank are likely to be healthier than the general population [77], and we excluded those with unusable OCT or MRI scans, which may result in selection bias. Second, the cross‐sectional nature of this study limits us from determining causality and temporality. Moreover, OCT and MRI scans were performed on different visits, and this time lapse may interfere with our results; however, we did adjust for this factor in the multivariable analysis to minimize its effect. OCT scans of the optic nerve head were not available in our dataset; peripapillary RNFL is possibly a more relevant measure than mRNFL.

In conclusion, thinner mRNFL, GCIPL, GCC, and total macular thicknesses are associated with smaller volumes of various subcortical brain structures and cortical regions, including the intracalcarine cortex, postcentral gyrus, cerebellum, thalamus, hippocampus, and basal ganglia. Notably, some of these relationships extend beyond anatomically established retina–brain connections. Findings from this in‐depth examination of the region‐specific associations between the retina and brain anatomy support the concept that retinal layer thicknesses are indices of regional brain structures.

## AUTHOR CONTRIBUTIONS

**Zihan Sun:** Visualization; writing – original draft; methodology; formal analysis; writing – review and editing. **Bing Zhang:** Conceptualization; methodology; writing – original draft. **Stephen Smith:** Methodology; writing – review and editing; supervision. **Denize Atan:** Writing – review and editing; supervision. **Anthony Khawaja:** Writing – review and editing; data curation; supervision; methodology. **Kelsey V. Stuart:** Writing – review and editing; data curation. **Robert N. Luben:** Data curation; writing – review and editing; project administration. **Mahantesh I. Biradar:** Writing – review and editing. **Thomas McGillivray:** Writing – review and editing. **Praveen Patel:** Writing – review and editing; data curation. **Peng T. Khaw:** Resources; writing – review and editing; funding acquisition. **Axel Petzold:** Writing – review and editing; project administration; supervision. **Paul Foster:** Conceptualization; supervision; resources; project administration; writing – review and editing; writing – original draft; funding acquisition.

## FUNDING INFORMATION

Z.S., P.J.F., A.P.K., R.N.L., P.T.K., P.J.P., and A.P. were supported by a grant from the National Institute for Health Research for a Biomedical Research Centre (BRC4) at Moorfields Eye Hospital NHS Foundation Trust and UCL (University College London) Institute of Ophthalmology. A.P.K. was supported by grants from UK Research and Innovation (UKRI) Future Leaders Fellowship, Alcon Research Institute Young Investigator Award, and Lister Institute Fellowship. R.N.L. was supported by UKRI Future Leaders Fellowship. P.T.K. was supported by grants from Helen Hamlyn Trust, Katz Foundation, Nolan Family, and Moorfields Eye Charity. K.V.S. is in receipt of a UCL Overseas Research Scholarship and is supported by grants from Fight for Sight (London) and the Desmond Foundation.

## CONFLICT OF INTEREST STATEMENT

D.A. sits on the clinical advisory board of Siloton. A.P.K. has received payment as a consultant to Abbvie, Aerie, Google Health, Novartis, Reichert, Santen, and Thea, and for lectures from Heidelberg Engineering outside the submitted work. P.J.P. has received payment as a consultant to Bayer UK, Genentech, Novartis, Roche UK, and Thea, and for lectures for Bayer and Roche. P.J.F. is recipient of an unrestricted grant from the Alcon Research Institute. P.J.F. has acted as a consultant to Alphasights, GLG, Google Health, Guidepoint, PwC, and Santen. P.T.K. has received payment as a consultant to Aerie, Alcon, Allergan, Belkin Vision, Novartis, Pfizer, Sanofi‐Aventis, Santen, and TwentyTwenty Therapeutics outside the submitted work. None of the other authors has any conflict of interest to disclose.

## DATA ACCESS, RESPONSIBILITY, AND ANALYSIS

Z.S. had full access to all the data in the study and takes responsibility for the integrity of the data and the accuracy of the data analysis.

## Supporting information


DATA S1.

DATA S2.

## Data Availability

The data that support the findings of this study are available from the corresponding author upon reasonable request.
