# Supplementary material for: Structural correlations between brain magnetic resonance image‐derived phenotypes and retinal neuroanatomy
Source: Eur J Neurol. 2024 May 8;31(7):e16288. doi: 10.1111/ene.16288 (PMC11235673; doi:10.1111/ene.16288)
Supplement: Supplementary file 1 — DATA S1. [file ENE-31-e16288-s002.docx]

ASSESSMENT OF COVARIABLES

**1. Age** was determined by subtracting the date of birth from the date of the OCT scan, representing the participant's age on the day of the ophthalmic assessment. It was then truncated to the nearest whole year (expressed as integers; Data-field ID 21003).

**2. Sex** was acquired from the central registry upon recruitment but may also include subsequent participant updates. Thus, this field can reflect both NHS-recorded and self-reported sex (Data-field ID 31).

**3. Imaging site:** The specific UK Biobank assessment centre where participants provided consent and underwent the brain imaging session (Cheadle, Reading, Newcastle, or Bristol; Data-field ID 54).

**4. Time lapse between OCT and brain MRI** was calculated by subtracting the age at the time of the brain MRI scan from the age on the day of the OCT scan, expressed as integers.

**5. Education level** was determined by the response to “Which of the following qualifications do you have (you can select more than one)?” (Options: college or university degree; A levels/AS levels or equivalent; O levels/GCSEs or equivalent; CSEs or equivalent; NVQ or HND or HNC or equivalent; other professional qualifications, e.g., nursing, teaching; none of the above). For analyses, qualifications were further categorized into three levels: (1) university degree; (2) A-level or professional qualifications; (3) O-level or less. The highest qualification across all 4 visits was used in the final analysis.

**6.** **Mean arterial pressure:** Systolic and diastolic blood pressure (Data-field IDs 4079, 4080) were measured twice at the assessment centre using an automated blood pressure device (Omron 705 IT electronic blood pressure monitor; OMRON Healthcare Europe B.V. Kruisweg 577 2132 NA Hoofddorp), or manually (Data-field IDs 93, 94) using a sphygmomanometer with an inflatable cuff in combination with a stethoscope if the blood pressure device failed to measure the blood pressure or if the largest inflatable cuff of the device did not fit around the participant's arm. All measurements were performed while the participant was sitting in a chair and were carried out by nurses trained in performing blood pressure measurements. Pulse pressure (PP) was calculated by subtracting the (average) diastolic from the (average) systolic blood pressure value. Mean arterial pressure (MAP) was calculated by dividing the PP by 3 and adding this value to the diastolic blood pressure. For analysis, we used the average MAP measurements across three visits – the initial assessment visit (2009-2010), the follow-up visit (2012-2013) and first imaging visit (2014+).

**7. Body mass index:** Weight and height were collected at baseline when participants attended the initial assessment centre. Height (cm) was measured using a Seca 202 device in all participants in the UK Biobank, along with sitting height. Weight (kg) was measured by a variety of means during the initial Assessment Centre visit, which was amalgamated into a single weight variable on the UK Biobank release data. Body mass index (BMI) was calculated as weight divided by height squared (kg/m^2^). For analysis, we used the average BMI measurements across three visits – the initial assessment visit (2009-2010), the follow-up visit (2012-2013) and first imaging visit (2014+).

**8. Smoking status** is determined by response to “Do you smoke tobacco now?” Options are “Current”, “Previous”, “Never”, and “Prefer not to answer”.

**9. Alcohol intake:** Information on habitual alcohol consumption was assessed in the baseline questionnaire (2006–2010). Participants were asked how often they drank alcohol and were required to categorize their response as: “Daily/almost daily”, “3–4 times a week”, “1–2 times a week”, “1–3 times a month”, “Special occasions only”, or “Never”. If their alcohol consumption varied substantially, participants were asked to provide an average considering their intake over the last year. Participants who reported a drinking frequency of “1–2 times a week” or greater were then asked to quantify their average weekly alcohol intake, whereas those reporting a frequency of “1–3 times a month” or “Special occasions only” were asked about their average monthly intake of each of the following: (1) “Glasses of red wine”; (2) “Glasses of white wine or champagne”; (3) “Pints of beer or cider”; (4) “Measures of spirits or liquors”; (5) “Glasses of fortified wine”; and (6) “Glasses of other [alcoholic drinks](https://www.sciencedirect.com/topics/medicine-and-dentistry/alcoholic-beverage).” These questions included definitions, examples, and standard portion sizes for each of the 6 alcoholic beverage types. Participants who reported a drinking frequency of “Never” to the first question were not asked to quantify their alcohol intake but were asked if they had previously drunk alcohol.

We then calculated average total alcohol (ethanol) intake (g/week) for all regular drinkers according to the formula:

$\sum_{i=1}^{6} {number of portions}_{\left( i \right)}$ ${portion size (mL)}_{(i)}$ ${alcohol concentration(g/mL)}_{(i)}$ *k*

where $i$ represents the alcoholic beverage categories described above and *k* represents a conversion factor depending on whether an individual reported their average weekly (*k*=1) or monthly (*k*=0.23) alcohol intake. For those reporting a weekly intake, the conversion factor does not change the quantitative estimate, whereas for those reporting a monthly intake, the conversion factor represents: (× 12 months/365 days × 7 days). The alcohol concentrations applied to each alcoholic beverage category were based on the same food composition tables and methodology used for the Oxford WebQ, a validated web-based [food frequency questionnaire](https://www.sciencedirect.com/topics/medicine-and-dentistry/food-frequency-questionnaire) that has been used to calculate alcohol intake in UK Biobank 24-hour dietary follow-up assessments.^1-3^ To handle implausibly low (e.g., regular drinkers reporting a weekly intake of 0 g) and extreme upper values, we excluded total alcohol intake estimates in the top and bottom 1 percentile. Full details of the derivation of alcohol intake from the UK Biobank questionnaire are described elsewhere.^4^

**10. Diabetes mellitus** is determined by those who answered "yes" to "Has a doctor ever told you that you have diabetes?"; or who answered "diabetic-related eye disease" to "Has a doctor told you that you have any of the following problems with your eyes?"; or who answered "insulin" to "Do you regularly take any of the following medications?”; or having self-reported non-cancer illness for diabetes (code:1220); or type 1 diabetes (code:1222); or type 2 diabetes (code:1223); or diabetic eye disease (code:1276); or diabetic neuropathy/ulcer (code:1468); or diabetic nephropathy (code: 1607).

**11. Spherical equivalent:** Refractive status for both eyes was assessed using autorefraction (Tomey RC5000; Erlangen-Tennenlohe). The spherical equivalent (SE) was derived as sphere + 0.5 × cylinder. Participant-level intraocular pressure (IOP) and spherical equivalent were determined as the mean of values from both eyes, if available. If data were present for only one eye, that value was used.

ASSESSMENT OF OTHER DEMOGRAPHIC, SYSTEMIC AND OCULAR CHARACTERISTICS

**1. IOPcc:** Intraocular pressure (IOP) measurements of ~115 000 participants in both eyes were taken in 2009–2010 using an Ocular Response Analyzer non-contact pneumotonometer (Reichert Corp., Philadelphia, PA, USA). Participants reporting an eye infection or surgery within the previous four weeks did not undergo IOP assessment. Individual-level IOP values were calculated as the mean of available right and left eye values, and extreme IOP values in the top and bottom 0.5 percentiles were excluded. This study used corneal-compensated IOP, a measure derived from a linear combination of inward and outward applanation tensions; it is least influenced by corneal biomechanical properties.

**2. Glaucoma:** Participants were considered glaucoma cases if they reported a diagnosis of glaucoma or previous surgical or laser treatment for glaucoma in either eye. We also excluded any participant carrying an International Classification of Diseases (ICD) code for glaucoma (ICD 9th revision: 365 [3651, 3652, 3653, 3654, 3655, 3656, 3658, 3659]; ICD 10th revision: H40 [H40.1, H40.2, H40.3, H40.4, H40.5, H40.6, H40.8, H40.9, H42.0, H42.8]) in their linked hospital records at any point prior to, and up to 1 year after, the baseline assessment.

**3. Townsend deprivation index** was derived from the participant's postcode at recruitment and the corresponding output area from the preceding national census. The index calculation incorporated factors such as employment status, home and car ownership, and household conditions. A higher, more positive index value indicates greater social deprivation (data-field ID 22189).

**4. Ethnicity:** Response options included: white (English/Irish or other white background), Asian or British Asian (Indian/Pakistani/Bangladeshi or other Asian background), black or black British (Caribbean, African, or other black background), Chinese, mixed (white and black Caribbean or African, white, and Asian, or other mixed background), or other ethnic groups (not defined)

**5. Use of anti-hypertension medications**: Participants were categorized as users of antihypertensive medications if they responded to the question, 'Do you regularly take any of the following medications (you can select more than one answer)?' and selected 'Blood pressure medication (code: 2).' Specific data-field IDs for women and man are 6153 and 6177, respectively.

STATISTICAL ANALYSIS

In the current report, our multivariable analysis involved performing 1530 pairwise linear regressions. Each regression model focused on examining the association between one specific retinal thickness measure and its corresponding brain IDP, one pair at a time, with the adjustment of a comprehensive set of covariates. To address potential Type I errors arising from multiple comparisons, we applied the Bonferroni correction, setting the significance threshold at P < 3.268×10^-5^. This correction method ensures stringent control over the familywise error rate, maintaining the overall Type I error rate at the desired level.

Alcohol intake quantity (g/week) demonstrated a right-skewed distribution and therefore underwent natural logarithm transformation to achieve a more normal distribution for subsequent statistical analyses. We employed a random forest approach to address missing data in the alcohol intake quantity (~7%) and education level (~4.4%) variables. This method was chosen due to its proficiency in managing mixed-type data and its resilience to outliers.^5^ The '*missForest*' package was utilized to estimate and replace the missing values.

SUPPLEMENTARY REFERENCES

1. Liu B, Young H, Crowe FL, et al. Development and evaluation of the Oxford WebQ, a low-cost, web-based method for assessment of previous 24 h dietary intakes in large-scale prospective studies. *Public health nutrition*. 2011;14(11):1998-2005.

2. Galante J, Adamska L, Young A, et al. The acceptability of repeat Internet-based hybrid diet assessment of previous 24-h dietary intake: administration of the Oxford WebQ in UK Biobank. *British Journal of Nutrition*. 2016;115(4):681-686.

3. Greenwood DC, Hardie LJ, Frost GS, et al. Validation of the Oxford WebQ online 24-hour dietary questionnaire using biomarkers. *American journal of epidemiology*. 2019;188(10):1858-1867.

4. Stuart KV, Luben RN, Warwick AN, et al. The association of alcohol consumption with glaucoma and related traits: findings from the UK Biobank. *Ophthalmology Glaucoma*. 2023;6(4):366-379.

5. Stekhoven DJ, Bühlmann P. MissForest—non-parametric missing value imputation for mixed-type data. *Bioinformatics*. 2012;28(1):112-118.
