# Supplementary material for: Structural correlations between brain magnetic resonance image‐derived phenotypes and retinal neuroanatomy
Source: Eur J Neurol. 2024 May 8;31(7):e16288. doi: 10.1111/ene.16288 (PMC11235673; doi:10.1111/ene.16288)
Supplement: Supplementary file 2 — DATA S2. [file ENE-31-e16288-s001.zip › ene16288-sup-0002-DataS2/ene16288-sup-0002-eTables_eFigures.docx]

**eTable 1. Brain Magnetic Resonance Image Derived Phenotypes and Corresponding UK Biobank Field IDs**

This includes 139 regional grey matter volumes and 15 subcortical structures' volumes (n=153).

*Abbreviations:* IDP=image derived phenotype

**eTable 2. Excluded Neurological Conditions**

Participants were excluded based on their self-reported data at the imaging visit where they underwent brain MRI scans.

**eTable 3. Univariate Pairwise Associations between Retinal Thicknesses and Brain Image Derived Phenotypes**

Bold indicates statistically significant associations (*P*<3.268×10^-5^); *r* represents Pearson’s correlation coefficient.

*Abbreviations:* IDP=image derived phenotype; R= right hemisphere; L=left hemisphere; CI=confidence interval; mRNFL=macular retinal nerve fiber layer; GCIPL=ganglion cell-inner plexiform layer; GCC=ganglion cell complex; INL=inner nuclear layer; INL-ELM=inner nuclear layer-external limiting membrane; INL-RPE=inner nuclear layer-retinal pigment epithelium; ELM-ISOS=external limiting membrane-inner segment outer segment; ISOS-RPE=inner segment outer segment-retinal pigment epithelium; RPE=retinal pigment epithelium.

**eTable 4.** **Multivariable Pairwise Associations between Retinal Thicknesses and Brain Image Derived Phenotypes**

Multivariable regression models were adjusted for age, sex, imaging site, time lapse between optical coherence tomography (OCT) and magnetic resonance imaging (MRI) scans, education level, mean arterial pressure, body mass index, smoking status, alcohol intake, diabetes, and spherical equivalence. Values in bold denote statistically significant associations with *P*<3.268×10^-5^. The presented *P* values are raw, not Bonferroni-corrected. Regression coefficients are given for every 1 µm change in retinal thickness corresponding to a 1 mm^3^ change in brain volumes. R-squared (%) values quantify the percentage of variance in a specific brain IDP explained by all the predictors in the model.

*Abbreviations:* IDP=image derived phenotype; R= right hemisphere; L=left hemisphere; CI=confidence interval; mRNFL=macular retinal nerve fiber layer; GCIPL=ganglion cell-inner plexiform layer; GCC=ganglion cell complex; INL=inner nuclear layer; INL-ELM=inner nuclear layer-external limiting membrane; INL-RPE=inner nuclear layer-retinal pigment epithelium; ELM-ISOS=external limiting membrane-inner segment outer segment; ISOS-RPE=inner segment outer segment-retinal pigment epithelium; RPE=retinal pigment epithelium.

**eTable 5. Partial R-Squared Values in Multivariable Regression Models between Retinal Thicknesses and Brain Image Derived Phenotypes**

Multivariable regression models were adjusted for age, sex, imaging site, time lapse between optical coherence tomography (OCT) and magnetic resonance imaging (MRI) scans, education level, mean arterial pressure, body mass index, smoking status, alcohol intake, diabetes, and spherical equivalence.

*Abbreviations:* IDP=image derived phenotype; R= right hemisphere; L=left hemisphere; GMV=grey matter volume; MAP=mean arterial pressure; BMI=body mass index; SE=spherical equivalence; mRNFL=macular retinal nerve fiber layer; GCIPL=ganglion cell-inner plexiform layer; GCC=ganglion cell complex; INL=inner nuclear layer; INL-ELM=inner nuclear layer-external limiting membrane; INL-RPE=inner nuclear layer-retinal pigment epithelium; ELM-ISOS=external limiting membrane-inner segment outer segment; ISOS-RPE=inner segment outer segment-retinal pigment epithelium; RPE=retinal pigment epithelium.

**eTable 6.** **Multivariable Pairwise Associations between Retinal Thicknesses and Brain Image Derived Phenotypes**

Multivariable regression models were adjusted for age, age^2^, sex, imaging site, time lapse between optical coherence tomography (OCT) and magnetic resonance imaging (MRI) scans, education level, mean arterial pressure, body mass index, smoking status, alcohol intake, diabetes, and spherical equivalence. Values in bold denote statistically significant associations with *P*<3.268×10^-5^. The presented *P* values are raw, not Bonferroni-corrected. Regression coefficients are given for every 1 µm change in retinal thickness corresponding to a 1 mm^3^ change in brain volumes. R-squared (%) values quantify the percentage of variance in a specific brain IDP explained by all the predictors in the model.

*Abbreviations:* IDP=image derived phenotype; R= right hemisphere; L=left hemisphere; CI=confidence interval; mRNFL=macular retinal nerve fiber layer; GCIPL=ganglion cell-inner plexiform layer; GCC=ganglion cell complex; INL=inner nuclear layer; INL-ELM=inner nuclear layer-external limiting membrane; INL-RPE=inner nuclear layer-retinal pigment epithelium; ELM-ISOS=external limiting membrane-inner segment outer segment; ISOS-RPE=inner segment outer segment-retinal pigment epithelium; RPE=retinal pigment epithelium.

**eTable 7.** **Multivariable Pairwise Associations between Retinal Thicknesses and Brain Image Derived Phenotypes**

Multivariable regression models were adjusted for age, sex, imaging site, time lapse between optical coherence tomography (OCT) and magnetic resonance imaging (MRI) scans, education level, mean arterial pressure, body mass index, smoking status, alcohol intake, diabetes, use of anti-hypertension medications, and spherical equivalence. Values in bold denote statistically significant associations with *P*<3.268×10^-5^. The presented *P* values are raw, not Bonferroni-corrected. Regression coefficients are given for every 1 µm change in retinal thickness corresponding to a 1 mm^3^ change in brain volumes. R-squared (%) values quantify the percentage of variance in a specific brain IDP explained by all the predictors in the model.

*Abbreviations:* IDP=image derived phenotype; R= right hemisphere; L=left hemisphere; CI=confidence interval; mRNFL=macular retinal nerve fiber layer; GCIPL=ganglion cell-inner plexiform layer; GCC=ganglion cell complex; INL=inner nuclear layer; INL-ELM=inner nuclear layer-external limiting membrane; INL-RPE=inner nuclear layer-retinal pigment epithelium; ELM-ISOS=external limiting membrane-inner segment outer segment; ISOS-RPE=inner segment outer segment-retinal pigment epithelium; RPE=retinal pigment epithelium.

**eTable 8.** **Multivariable Pairwise Associations between Retinal Thicknesses and Brain Image Derived Phenotypes**

In the sensitivity analysis, participants with a self-reported history of multiple sclerosis, Parkinson's disease, Alzheimer's disease, dementia, cognitive impairment, stroke, and ischemic stroke were excluded. Multivariable linear regressions were conducted using data from a cohort of n=6,362 participants. The regressions were adjusted for the following covariates: age, sex, imaging site, time lapse between optical coherence tomography (OCT) and magnetic resonance imaging (MRI) scans, education level, mean arterial pressure, body mass index, smoking status, alcohol intake, presence of diabetes mellitus, and spherical equivalence. Values in bold denote statistically significant associations with *P*<3.268×10^-5^. The presented *P* values are raw, not Bonferroni-corrected. Regression coefficients are given for every 1 µm change in retinal thickness corresponding to a 1 mm^3^ change in brain volumes. R-squared (%) values quantify the percentage of variance in a specific brain IDP explained by all the predictors in the model.

*Abbreviations:* IDP=image derived phenotype; R= right hemisphere; L=left hemisphere; CI=confidence interval; mRNFL=macular retinal nerve fiber layer; GCIPL=ganglion cell-inner plexiform layer; GCC=ganglion cell complex; INL=inner nuclear layer; INL-ELM=inner nuclear layer-external limiting membrane; INL-RPE=inner nuclear layer-retinal pigment epithelium; ELM-ISOS=external limiting membrane-inner segment outer segment; ISOS-RPE=inner segment outer segment-retinal pigment epithelium; RPE=retinal pigment epithelium.

**eFigure 1. Study Flow Diagram.**

*To clarify, for participants without available OCT images at the baseline visit (2009-2010), we utilized the OCT data from their follow-up visit (2012-2013) for analysis.

*Abbreviations:* OCT=optical coherence tomography; MRI= magnetic resonance imaging; IOP=intraocular pressure; VA=visual acuity; logMAR=logarithm of the minimal angle of resolution; CNS=central nervous system.

**eFigure 2. Visual Representation of the Results of Pairwise Multivariable Association Tests Between 10 Retinal Metrics and 153 Brain Magnetic Resonance Image Derived Phenotypes (IDPs).**

Multivariable linear regressions were performed using data from n=6,421 participants, adjusting for age, age^2^, sex, imaging site, the time lapse between OCT and MRI scan, education level, mean arterial pressure, body mass index, smoking status, alcohol intake, diabetes mellitus, and spherical equivalence. Each datapoint represents a single retina-brain association: blue circles indicate positive regression coefficients; orange squares indicate negative regression coefficients. We followed the convention for Manhattan plots and plotted -log10 (*P* values) on the y-axis. The dashed horizontal line indicates the -log10 (*P*) threshold after Bonferroni correction is applied, and all associations above this line are considered statistically significant at P<3.268×10^-5^ (corresponding to a -log10 (*P*) of 4.4857).

*Abbreviations:* mRNFL=macular retinal nerve fiber layer; GCIPL=ganglion cell-inner plexiform layer; GCC=ganglion cell complex; INL=inner nuclear layer; INL-ELM=inner nuclear layer-external limiting membrane; INL-RPE=inner nuclear layer-retinal pigment epithelium; ELM-ISOS=external limiting membrane-inner segment outer segment; ISOS-RPE=inner segment outer segment-retinal pigment epithelium; RPE=retinal pigment epithelium; Total=total macular thickness; GM=grey matter; R= right hemisphere; L=left hemisphere.

**eFigure 3. Alluvial Diagram Illustrating the Statistically Significant Retina-Brain Associations Based on Vascular Supply Territories.**

For clarity, only statistically significant associations identified by multivariable regression of mRNFL and GCIPL are shown in this diagram. Covariates adjusted in these multivariable models include age, sex, imaging site, the time lapse between OCT and MRI scans, education level, mean arterial pressure, body mass index, smoking status, alcohol intake, diabetes mellitus, and spherical equivalence. Each stream in the diagram corresponds to a datapoint (retina-brain association) shown in Figure 3 falling above the Bonferroni-corrected threshold line. The width of each stream is proportional to the partial R-squared, as shown in Table 2. In the right block, anterior represents structures supplied by the anterior cerebral artery; anterior/middle represents structures supplied by both anterior and middle cerebral artery; middle represents structures supplied by the middle cerebral artery; posterior represents structures supplied by the posterior cerebral artery.

*Abbreviations:* mRNFL=macular nerve fibre layer; GCIPL=ganglion cell-inner plexiform layer; GM=grey matter; R= right hemisphere; L=left hemisphere.
