# Supplementary figures and images for: Structural correlations between brain magnetic resonance image‐derived phenotypes and retinal neuroanatomy
Source: Eur J Neurol. 2024 May 8;31(7):e16288. doi: 10.1111/ene.16288 (PMC11235673; doi:10.1111/ene.16288)

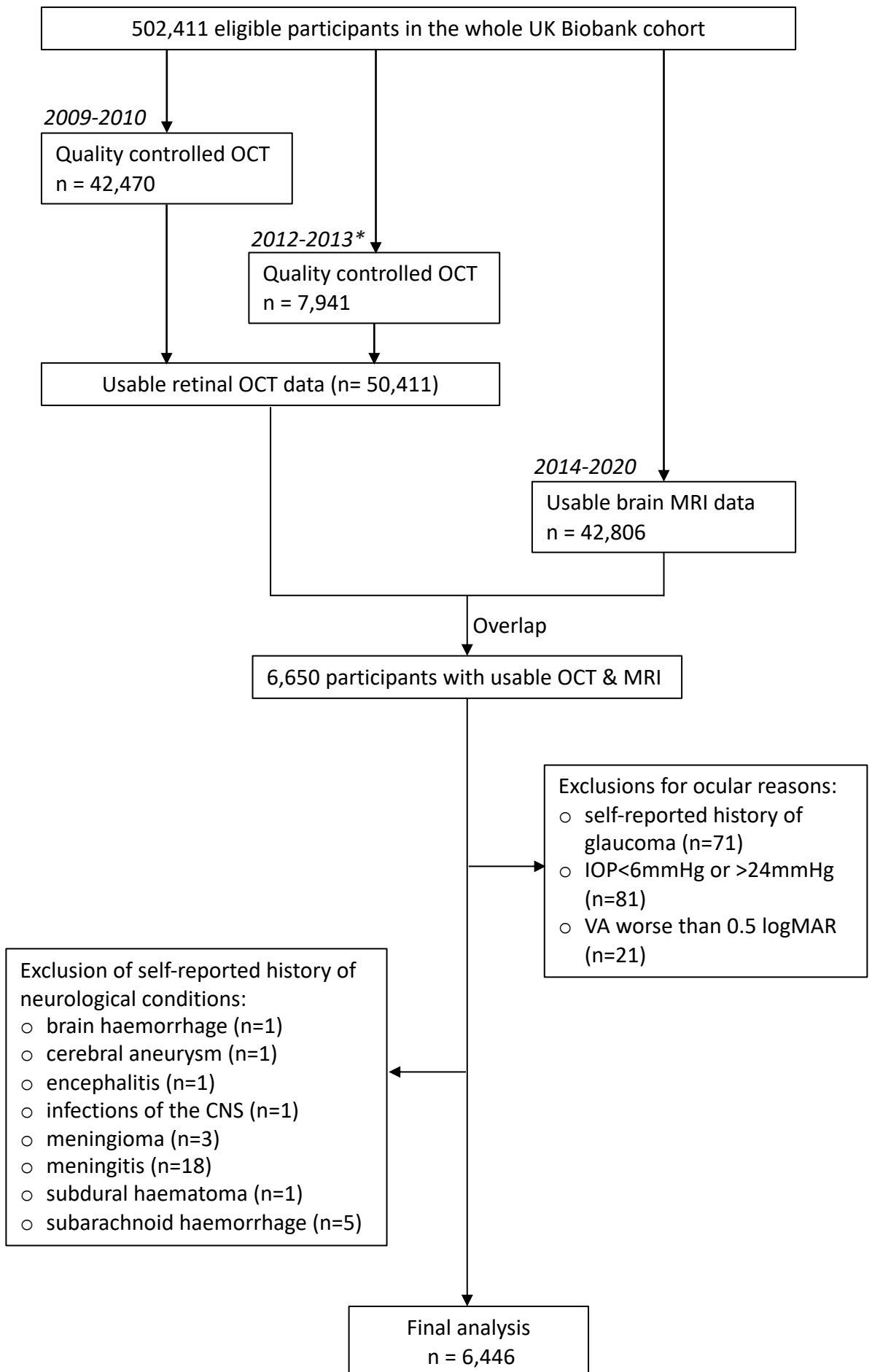

Supplement: Supplementary file 2 — DATA S2. [file ENE-31-e16288-s001.zip › ene16288-sup-0002-DataS2/eFigure_1_20240130.pdf]

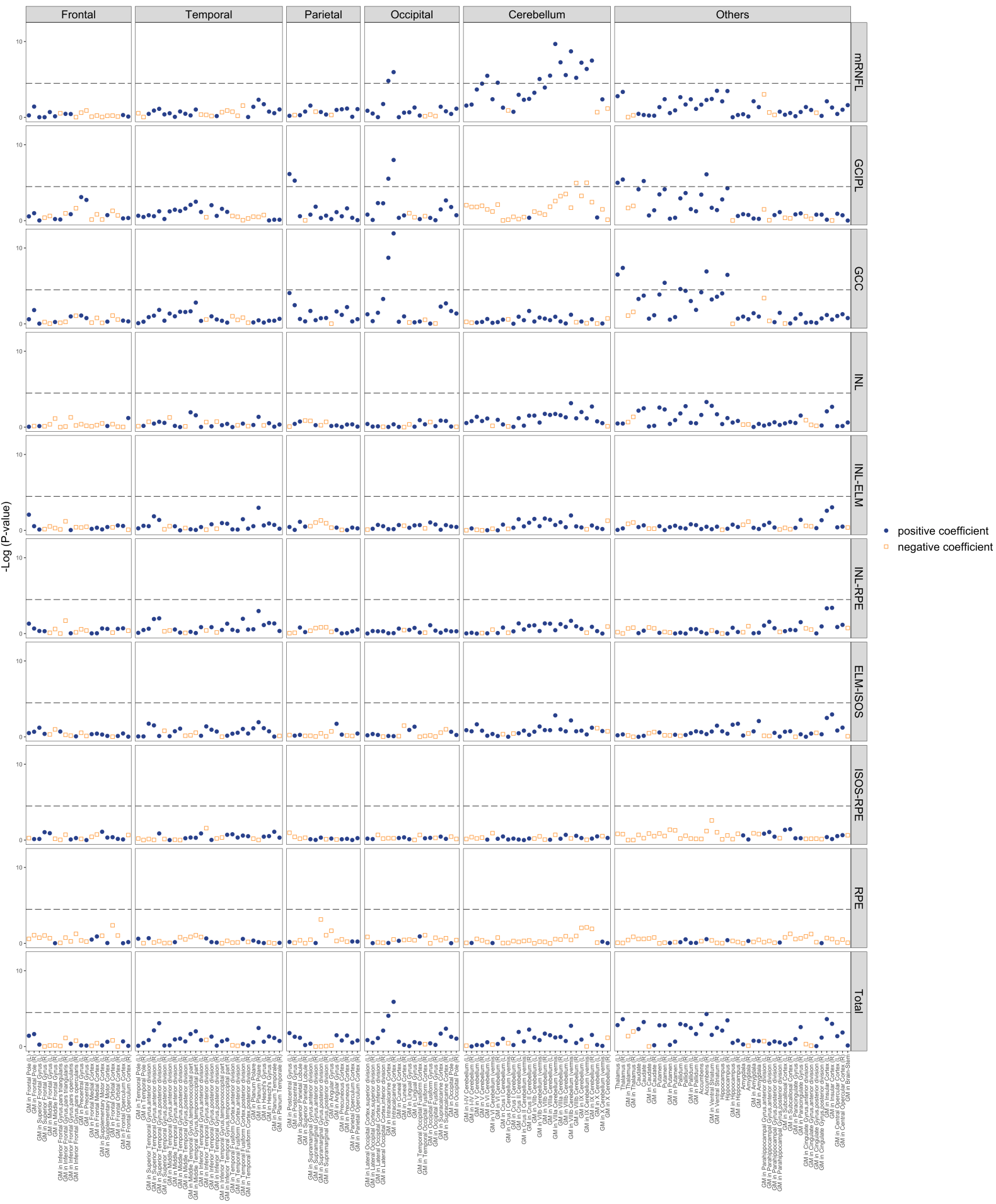

Supplement: Supplementary file 2 — DATA S2. [file ENE-31-e16288-s001.zip › ene16288-sup-0002-DataS2/eFigure_2_20240130.pdf]

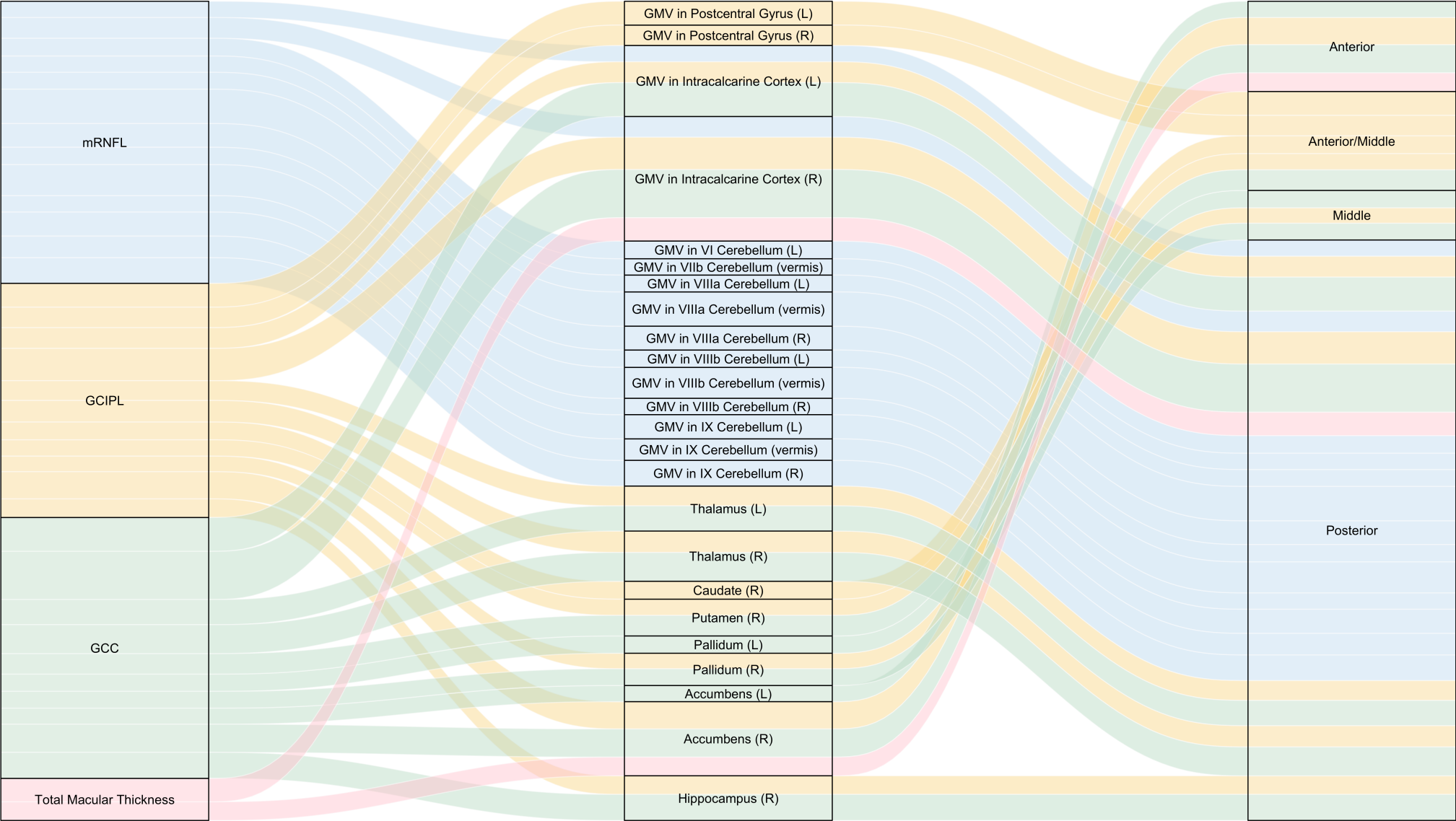

Supplement: Supplementary file 2 — DATA S2. [file ENE-31-e16288-s001.zip › ene16288-sup-0002-DataS2/eFigure_3_20240201.pdf]
